# The Effects of Mindfulness-Based Stress Reduction on Trauma in Victims of Gun Violence: a Pilot Study

**DOI:** 10.1007/s12671-022-01858-y

**Published:** 2022-03-22

**Authors:** Lora Khatib, Gabriel Riegner, Jon G. Dean, Valeria Oliva, Gael Cruanes, Beth A. Mulligan, Fadel Zeidan

**Affiliations:** 1 Department of Anesthesiology, University of California San Diego, 9500 Gilman Drive, MC 0719, La Jolla, CA 92093 USA; 2 Mindful Way, Mindfulness-Based Training Programs, Banning, USA

**Keywords:** Mindfulness-based stress reduction, Gun violence, Trauma, Grief, Depression

## Abstract

**Objectives:**

Gun violence is a significant problem in the United States of America. Gun violence produces lifelong psychological adversity, trauma, and grief. In the face of this epidemic, efficacious therapies that assuage gun violence-based trauma and negative health are lacking.

**Methods:**

The proposed, longitudinal pilot experiment examined the effects of an 8-week mindfulness-based stress reduction (MBSR) program on traumatized individuals as a direct consequence of gun violence. Twenty-four victims of gun violence (median age = 53 years; 21 female) completed measures of the primary outcome: trauma. Secondary outcomes were characterized as grief, depression, sleep quality, life satisfaction, and mindfulness. All assessments were administered before, after 5, and 8 weeks of MBSR training. It was hypothesized that trauma and other comorbidities would improve following MBSR. It was also predicted that outcomes would be significantly stronger from baseline to 5 weeks of MBSR training than from 5 to 8 weeks of training.

**Results:**

Before MBSR, volunteers exhibited high levels of trauma, depression, sleep difficulty, and grief. Participation in MBSR was associated with improved trauma, depression, sleep difficulty, and life satisfaction. The most pronounced improvements in psychological disposition were exhibited within the first 5 weeks of MBSR. However, these benefits were largely preserved after completion of the course. Importantly, increases in dispositional mindfulness predicted lower trauma, complicated grief, and sleep difficulties.

**Conclusions:**

The present findings should be interpreted with caution because they were derived from an uncontrolled, non-randomized trial. However, said findings suggest that MBSR may reduce trauma and improve overall well-being in gun violence victims.

Gun violence is an American public health epidemic that has catastrophic consequences on individual and societal well-being. In this study, gun violence is characterized as the assault on individuals using firearms. This includes firearm-based homicide, violent crime, attempted suicide, suicide, and unintentional death and injury. According to the Centers for Disease Control and Prevention (2021), gun violence was the leading cause of death for Black youth and the second leading cause of death for American youth in 2019. Over 39,000 Americans were killed by a gun in 2019 and over 30,000 were left injured. In 2020, said figure increased to over 43,000 deaths caused by firearms with over 30,000 injured (Gun Violence Archive, 2021). Gun violence costs the United States of America’s (USA) economy approximately $229 billion annually in medical treatments, family care, judicial expenditures, loss of income, and daily care/support. Furthermore, firearm-based mass shootings, suicide, domestic violence, and unintentional deaths have risen dramatically in the past 20 years, causing the USA to have the highest rate of mortality from firearms amongst other developed nations (Centers for Disease Control and Prevention, 2021; Grinshteyn & Hemenway, 2016; Krouse & Richardson, 2015). Gun violence has become so prominent in the USA that the likelihood of knowing a victim of gun violence within any individual’s network is 99.9% (Kalesan et al., 2016).

Despite the vast prevalence of this epidemic, research on gun violence is scarce when compared to other leading causes of death (Stark & Shah, 2017). However, there is extant research demonstrating that mass shootings produce a high occurrence of posttraumatic stress and depression (Bardeen et al., 2013; Hawdon et al., 2012; Littleton et al., 2009; Lowe & Galea, 2015; North et al., 2001; Séguin et al., 2013; Smith et al., 2015; Suomalainen et al., 2011; Vicary & Fraley, 2010). Although the lasting, detrimental effects of mass shootings have been made apparent, these events only account for less than 2% of gun-related deaths a year (Gun Violence Archive, 2021). The limited number of studies that have examined the psychological effects of individuals exposed to gun violence fatalities, not specific to mass-shootings, have underscored the unique, deleterious consequences that arise from the sudden and maliciously violent nature of these events.

A recent study found that individuals exposed to gun violence fatalities are significantly more likely to suffer from psychological distress, depression, suicidal ideation, and psychotic-like experiences (Smith et al., 2020). Another study revealed that the death of a loved one from a shooting led to high levels of posttraumatic stress and grief, and the severity of posttraumatic stress predicted persistent grief (Smith et al., 2015). Taken together, these findings reveal that victims of gun-violence are at high risk for severe posttraumatic stress, persistent grief, and depression. However, no study to date has identified effective interventions to ameliorate the coping and grieving process for gun violence victims.

Mindfulness-based stress reduction (MBSR), an 8-week evidence-based program designed to treat chronically ill patients that “have fallen through the cracks,” may be advantageous for individuals who are suffering from a traumatic experience caused by gun violence (Kabat-Zinn, 1982). MBSR provides intensive training on nonjudgmental reactivity to positive and stressful sensory events by focusing attention on somatic sensations (breath, body) through mindfulness practices such as “body scan” (i.e., nonjudgmental focus on different parts of the body) and mindful awareness of breath (Fischer et al., 2017; Sauer-Zavala et al., 2013). MBSR improves a variety of mental and physical health outcomes including stress, depression, anxiety, and chronic pain in both clinical and nonclinical settings (Biegel et al., 2009; Chiesa & Serretti, 2009; Hazlett-Stevens, 2012; Khoury et al., 2015; Rosenzweig et al., 2010; Serpa et al., 2014). More recently, MBSR has been used to treat symptoms of trauma that are not related to gun violence.

It was recently demonstrated that MBSR significantly reduced PTSD symptoms in 14 individuals that suffered from traumatic stress in response to car accidents, child abuse, and a spectrum of other disturbing events (Müller-Engelmann et al., 2017). Additionally, recent randomized, controlled trials examining the effects of MBSR on veterans suffering from PTSD found that MBSR was more effective than present-centered group therapy (PCGT), an intervention specifically tailored to treating trauma, at reducing PTSD symptomology (Davis et al., 2019; Polusny et al., 2015). Still, the trauma that arises from grieving the death of a loved one to gun violence may be more complicated than other forms of trauma as the unanticipated and cruel nature of these circumstances often leads to other adverse symptoms along with trauma, such as intense grief, a loss of trust in humanity, and a loss of meaning in oneself and the world around them (Armour, 2003; Bailey et al., 2013). Thus, whether MBSR can improve trauma and other psychological outcomes that arise as a result of the death of a loved one from gun violence is an open question.

It is evident that the alleviation of trauma and suffering in the growing number of gun violence victims in the USA is needed. The primary aim of the present pilot study is to examine whether participation in MBSR is associated with reductions in trauma and improvements in overall well-being in individuals who experienced a traumatic event caused by gun violence. The secondary aim of this study is to investigate whether the benefits associated with MBSR change as a function of training dosage. Exploratory regression analyses tested whether dispositional mindfulness increases after MBSR and if increased dispositional mindfulness is predictive of improvements in trauma and corresponding comorbidities.

## Methods

### Participants

Twenty-four volunteers (median age = 53 years; 21 female) were recruited and screened by “Survivors Empowered,” a non-profit organization that provides support and referrals for survivors of gun violence. Survivors Empowered also provides victims of gun violence with a social support network, and a “safe space” to share stories and collaborate on ways to reduce gun violence. All recruited participants reported experiencing daily trauma and grief directly as a cause from gun violence. Twenty-three participants lost an immediate family member to a gun and one participant was shot himself (Table 1).

**Table 1:** Participant demographics

Gender	*N*	%
Male	3	12.5%
Female	21	87.5%
Ethnicity	*N*	%
White	18	75.0%
Black or African American	5	20.8%
Other	1	4.2%
Relationship status	*N*	%
Married	20	83.3%
Divorced	2	8.3%
Single	2	8.3%
Highest level of education	*N*	%
High school	7	29.2%
Associate’s	6	25.0%
Bachelor’s	5	20.8%
Master’s	4	16.7%
Professional	2	8.3%

Self-reported demographics of study population (*N* = 24). During data collection, participants resided in Arizona, California, Colorado, Connecticut, Florida, Illinois, Indiana, Kentucky, Maryland, Michigan, New York, New Jersey, Nevada, South Carolina, and Texas

### Procedure

**Fig. 1:**
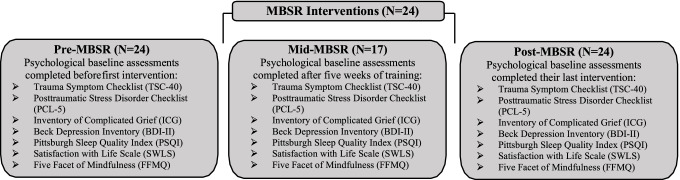
Assessments were administered 1 week before MBSR courses started, after the completion of five courses but before the daylong retreat, and after completion of the course. The Inventory of Complicated Grief had less responses due to the option to opt out of this survey (*N* = 13 for pre- and post-MBSR; *N* = 9 for mid-MBSR)

The proposed research activities were approved by the UCSD Institutional Review Board (IRB#192,007) and were conducted online during the global novel coronavirus (COVID-19) pandemic (January 4th, 2021, to April 6th, 2021). Participants were screened and enrolled into the proposed project by the study team. Prior to providing informed consent, participants completed pre-intervention assessments (pre-MBSR). Participants then attended five, 2.5-h weekly MBSR classes via the videoconferencing platform, Zoom (Zoom Video Communications Inc., 2019) (Fig. 1). Participants then completed assessments at 5 weeks (mid-MBSR). After 8 weeks of MBSR, participants completed post-intervention assessments (post-MBSR). Seven of the twenty-four participants did not complete mid-MBSR assessments. Biological sample kits were also administered but only 5 participants completed these assessments. Thus, biological data are not presented here.

#### Mindfulness-Based Stress Reduction

All subjects participated in a standard 8-week MBSR course and there were no explicit didactics tailored to addressing trauma and grief. MBSR consisted of eight 2.5-h weekly classes. After 5 weeks of MBSR, subjects participated in a daylong (7 h) silent meditation retreat. The course was taught in two separate cohorts by four ethnically diverse, certified mindfulness teachers who closely followed the guidelines described by Kabat-Zinn (1982). Two trauma-informed therapists were also present but did not explicitly interact with participants. Therapists attended MBSR sessions in case of any adverse events.

The aim of MBSR was to provide participants with formalized mindfulness didactics to better incorporate mindfulness-based coping strategies and direct modulation of appraisals of discursive sensory, cognitive, and affective events. Participants were taught to attend to the present moment, engaging a number of somatic (body, breathing) and ruminations non-reactively. In addition to the spectrum of guided mindfulness-based practices (e.g., sitting meditation, body scan, and Hatha Yoga) introduced in the first 4 weeks of the course, the last 4 weeks also included real-life applications of mindfulness such as emotional regulation and compassion for others. Participants were also provided daily assignments to be performed outside of the formal training. To promote adherence and compliance to MBSR, study participants were provided workbooks, audiobooks, and explicit instructions to practice meditation outside of MBSR class.

### Measures

A battery of psychometrically validated questionnaires was employed to determine if MBSR is associated with improved well-being in victims of gun violence. All assessments were delivered and automatically scored using REDCap (Research Electronic Data Capture; Harris et al., 2009). All research technicians were blinded to self-report scales until study completion.

#### Trauma Symptom Checklist-40 (TSC-40)

The Trauma Symptom Checklist-40 (TSC-40) is a widely used 40-item self-report scale that assessed the frequency in which distressing symptoms arise from past traumatic experiences (Elliott & Briere, 1992). The TSC-40 consists of six subscales: dissociation, anxiety, depression, trauma history, sleep disturbances, and sexual problems. Subscale data are not presented here. Higher scores indicated higher frequency of trauma symptoms. The internal consistency of this scale in the present sample ranged from good to excellent (Cronbach, 1951; pre-MBSR: Cronbach’s *α* = 0.89, McDonald’s *ω* = 0.90; mid-MBSR: *α* = 0.91, *ω* = 0.94; post-MBSR: *α* = 0.90, *ω* = 0.93).

#### PTSD Checklist-5 (PCL-5)

The PTSD Checklist-5 (PCL-5; Weathers et al., 2013) is a 20-item self-report measure that evaluated the severity of one’s experience with the 20 *DSM-5* PTSD symptoms (e.g., repeated, disturbing, and unwanted memories of the stressful experience) in the past month. Higher scores indicated higher PTSD. The reliability of this scale in the current sample was excellent (pre-MBSR: *α* = 0.93, *ω* = 0.96; mid-MBSR: *α* = 0.90, *ω* = 0.94; post-MBSR: *α* = 0.91, *ω* = 0.95).

#### Inventory of Complicated Grief

The Inventory of Complicated Grief (ICG; Prigerson et al., 1995) is a 19-item self-report scale that measured pathological grief. Participants were asked to complete the ICG in reference to a loved one they have lost due to gun violence. A warning and an option to opt out of this survey was given to participants due to the potentially triggering nature of this survey. Consequently, 11 participants opted out of completing this scale. The ICG demonstrated excellent internal consistency in the present sample (pre-MBSR: *α* = 0.94, *ω* = 0.97; mid-MBSR: *α* = 0.96, *ω* = 0.98; post-MBSR: *α* = 0.96, *ω* = 0.98).

#### Beck Depression Inventory-II

The Beck Depression Inventory-II (BDI-II; Beck et al., 1996) is a 21-item assessment that measured depressive symptomology, mood disturbance, negative affect, and depressive mood. Higher scores indicated greater levels of depressive symptomology/mood (Adler-Neal et al., 2019). Reliability of this scale ranged from good to excellent (pre-MBSR: *α* = 0.90, *ω* = 0.93; mid-MBSR: *α* = 0.90, *ω* = 0.94; post-MBSR: *α* = 0.89, *ω* = 0.94).

#### Pittsburgh Sleep Quality Index

The Pittsburgh Sleep Quality Index (PSQI) contains 19 self-report items that assessed quality of sleep (Buysse et al., 1989). The PSQI consists of seven “component” scores including subjective sleep quality, sleep latency, sleep duration, habitual sleep efficiency, sleep disturbances, use of sleeping medication, and daytime dysfunction. Subscale data are not presented here. Higher scores indicated more severe sleep difficulties. Internal consistency of this scale ranged from acceptable to good (pre-MBSR: *α* = 0.79, *ω* = 0.85; mid-MBSR: *α* = 0.76, *ω* = 0.87; post-MBSR: *α* = 0.82, *ω* = 0.83).

#### Satisfaction with Life Scale

The Satisfaction with Life Scale (SWLS) is a 5-item scale that was used to assess one’s holistic life satisfaction (Diener et al., 1985). Higher scores indicated higher life satisfaction. The SWLS demonstrated good reliability in the current sample (pre-MBSR: *α* = 0.86, *ω* = 0.91; mid-MBSR: *α* = 0.84, *ω* = 0.92; post-MBSR: *α* = 0.89, *ω* = 0.94).

#### Five Facet Mindfulness Questionnaire

Hypothesized changes in dispositional mindfulness were measured using the Five Facet Mindfulness Questionnaire (FFMQ; Baer et al., 2006). The FFMQ consists of five subscales: observation, description, aware actions, non-judgment, and non-reactivity. Subscales are not presented here. Higher scores represented higher levels of trait mindfulness. Internal consistency of this scale was excellent (pre-MBSR: *α* = 0.91, *ω* = 0.95; mid-MBSR: *α* = 0.94, *ω* = 0.97; post-MBSR: *α* = 0.94, *ω* = 0.96).

### Data Analyses

Paired samples *t*-tests (SPSS version 26.0) examined if there were significant improvements before and after MBSR in all measures (i.e., trauma symptoms (TSC-40), posttraumatic stress (PCL-5), grief (ICG), depression (BDI-II), sleep difficulty (PSQI), life satisfaction (SWLS), and dispositional mindfulness (FFMQ)), respectively. All *t*-tests were Bonferroni corrected for multiple comparisons (*p* < 0.007). Seventeen of the twenty-four participants completed assessments for all three time points (Fig. 1). Repeated measures ANOVAs examined if there were significant changes in outcomes from before to the middle (5 weeks) and after MBSR (after 8 weeks). A priori simple effects tests were performed to interpret significant main effects. The repeated measures ANOVA reflected listwise deletion in 7 individuals. Thus, the results from the repeated measures ANOVA correspond to 17 individuals. Exploratory simple linear regressions were computed to test if improvements in dispositional mindfulness from pre- to post-MBSR predicted changes in (a) trauma (TSC), (b) posttraumatic stress (PCL-5), (c) grief (ICG), (d) depression (BDI-II), (e) sleep (PSQI), and (f) satisfaction with life (SWLS).

## Results

### Summary of Psychological Outcomes at Baseline

At baseline (pre-MBSR), 38% of participants met the criteria that is indicative of probable PTSD (PCL-5; Department of Veteran Affairs, 2015). Seventy-nine percent (79%) of participants scored above the cutoff point on complicated grief that is considered “at high risk for requiring clinical care” (ICG; Prigerson et al., 1995). Thirty-eight percent (38%) of participants also met the criterion for clinical depression (Beck et al., 1996). Seventy-nine (79%) of participants scored above the standardized cutoff point that distinguishes a “poor” sleeper (PSQI; Buysse et al., 1991).

### The Relationship Between MBSR, Trauma, and Other Comorbidities

Participants reported a 37% reduction in trauma [TSC-40; *t*(23) =  − 5.38, *p* < 0.001, Cohen’s *d* (*d*) =  − 1.10] and 52% reduction in posttraumatic stress [PCL-5; *t*(23) =  − 4.71, *p* < 0.001, *d* =  − 0.96] after 8 weeks of MBSR. MBSR was also associated with a 52% reduction in depression [BDI-II; *t*(23) =  − 5.50, *p* < 0.001, *d* =  − 1.12]. Grief decreased by 23% [ICG; *t*(12) =  − 2.83, *p* = 0.02, *d* =  − 0.78]; however, this effect did not survive Bonferroni correction. Sleep difficulties were significantly reduced by 26% from pre- to post-MBSR [PSQI; *t*(23) =  − 3.81, *p* = 0.001, *d* =  − 0.78], and participants reported an increase in overall life satisfaction by 16% [SWLS; *t*(23) = 2.93, *p* = 0.008, *d* = 0.60].

### The Relationship Between MBSR Training Dosage, Trauma, and Other Comorbidities

**Fig. 2:**
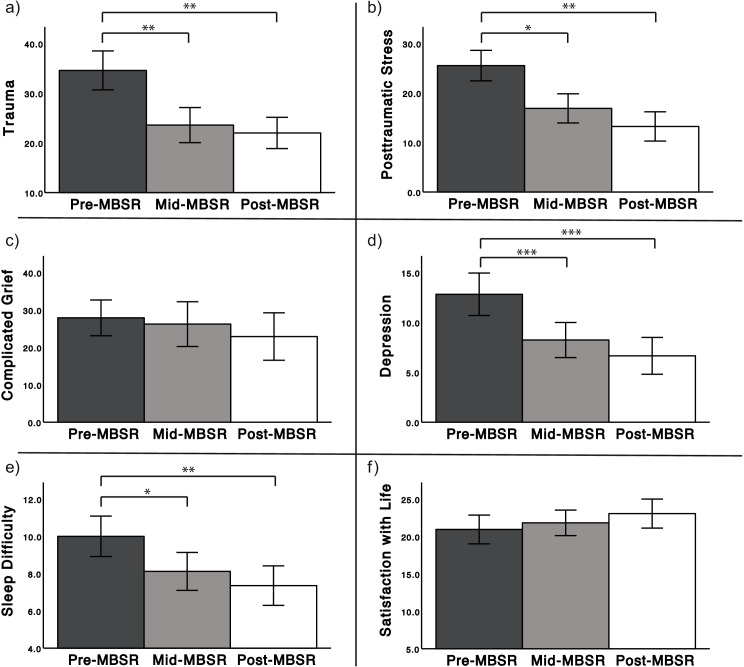
a) Trauma significantly decreased from pre- to mid-, and pre- to post-MBSR (TSC-40; *N* = 17). b) Posttraumatic stress significantly decreased from pre- to mid-, and pre- to post-MBSR (PCL; *N* = 17). c) Complicated grief reductions were not significant (ICG; *N* = 9). d) Depression significantly decreased from pre- to mid-, and pre- to post-MBSR (BDI-II; *N* = 17). e) Sleep difficulty significantly decreased from pre- to mid-, and pre- to post-MBSR (PSQI; *N* = 17). f) Life satisfaction increases were not significant (SWLS; *N* = 17), ^*^p ≤ .05, ^**^p ≤ .01, ^***^p ≤ .001. Error bars: ± 1 SE

A significant main effect of time on self-reported trauma was observed [*F*(2, 16) = 10.10, *p* = 0.002; Fig. 2a] that was driven by the significant reductions from pre- to mid-MBSR and from pre- to post-MBSR (*p* ≤ 0.001), respectively. There was no significant change in trauma from mid- to post-MBSR (*p* = 0.42). The significant main effect on posttraumatic stress [*F*(2, 16) = 8.64, *p* = 0.002; Fig. 2b] was a result of the significant reductions from pre- to mid- (*p* < 0.05), and pre- to post-MBSR (*p* = 0.001), but not from mid- to post-MBSR (*p* = 0.11).

Similarly, the significant main effect of depression [*F*(2, 16) = 17.02, *p* < 0.001; Fig. 2d] was associated with significant decreases from pre- to mid- and pre- to post-MBSR (*p* ≤ 0.001), but not mid- to post-MBSR (*p* = 0.12). Changes in sleep difficulties [*F*(2, 16) = 7.00, *p* = 0.003; Fig. 2e] were also significant across time. This effect was driven by significant increases from pre- to mid- (*p* = 0.02) and pre- to post-MBSR (*p* = 0.005), but not mid- to post-MBSR (*p* = 0.24). There were no significant changes in grief (*p* = 0.140; Fig. 2c) or life satisfaction (*p* = 0.180; Fig. 2f).

### The Relationship Between Dispositional Mindfulness and Well-being

**Fig. 3:**
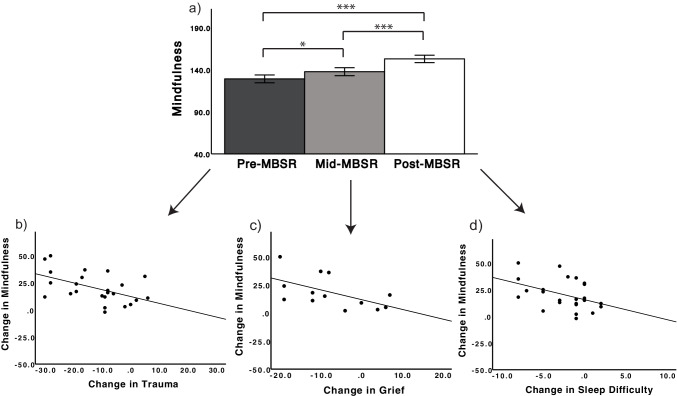
a) Dispositional mindfulness significantly increased from pre- to mid-, mid- to post-, and pre- to post-MBSR (FFMQ; *N* = 17), ^*^p ≤ .05, ^**^p ≤ .01, ^***^p ≤ .001. Error bars: ± 1 SE. Changes (post–pre) in mindfulness predicted changes (post–pre) in b) trauma (TSC-40; *R*^2^ = 0.26), c) grief (ICG; *R*^2^ = 0.31), d) and sleep difficulties (PSQI; *R*^2^ = 0.18)

As predicted, dispositional mindfulness significantly increased by 15% after MBSR [FFMQ; *t*(23) = 7.10, *p* < 0.001, *d* = 1.45]. The main effect of time on dispositional mindfulness scores [*F*(2, 16) = 27.67, *p* < 0.001; Fig. 3a] demonstrated a significant linear increase across training stages (pre- to mid-: *p* = 0.03, pre- to post-: *p* < 0.001, and mid- to post-MBSR: *p* < 0.001). Heightened dispositional mindfulness predicted greater reductions in (a) trauma symptoms [*F*(1, 23) = 7.67, *p* = 0.01, *R*^2^ = 0.26; Fig. 3b], (b) grief [*F*(1, 12) = 4.95, *p* = 0.048, *R*^2^ = 0.31; Fig. 3c], and (c) sleep difficulties [*F*(1, 23) = 4.69, *p* = 0.04, *R*^2^ = 0.18; Fig. 3d]. Mindfulness was not significantly associated with improvements in posttraumatic stress (*p* = 0.12), depression (*p* = 0.54), or satisfaction with life (*p* = 0.27).

## Discussion

In this pilot study, we investigated whether trauma and associated psychological outcomes in gun violence victims were improved after MBSR. Not surprisingly, the present sample of gun violence victims reported frequent and severe symptoms of trauma and posttraumatic stress compared to averages reported in the literature of individuals who suffered from abuse or a stressful life event (Blevins et al., 2015; Neal & Nagle, 2013). This sample also reported high complicated grief compared to non-gun-related bereaved individuals (Prigerson et al., 1995). Depression and sleep difficulties were also high compared to healthy samples (Buysse et al., 1991; von Glischinski et al., 2019). Nevertheless, MBSR was associated with meaningful improvements across these outcomes. Participants also reported an increase in dispositional mindfulness and satisfaction with life following MBSR. Improvements in mindfulness predicted lower trauma, complicated grief, and sleep difficulties.

Notably, 8 weeks of MBSR training were associated with significantly improved scores on all measures, except complicated grief, with medium to large effect sizes observed for all measures. Interestingly, the rate of improvement was much faster and stronger in the first 4 weeks of MBSR, when compared to the latter half. These findings are consistent with previous work demonstrating high efficacy after brief mental training (Possemato et al., 2016; Quaglia et al., 2019; Zeidan et al., 2010). Yet, the improvements were largely sustained and improved upon with more frequent training, providing supplementary evidence that mindfulness training is analogous to physical training and can produce long lasting, stabilized improvements in psychological disposition. What is unclear is if these sustained improvements, during the intervention, were attributable to non-specific effects such as social support, facilitator attention, and demand characteristics. Nevertheless, the improvements in psychological disposition are quite profound considering the high level of trauma and stress this particular population faces on a daily basis. Only thirteen of the twenty participants completed the Inventory of Complicated Grief due to the “triggering” nature of the scale. Thus, the 23% improvement in grief from MBSR did not survive Bonferroni correction. More work is needed to determine if MBSR-induced grief reductions are sustainable when compared to more active therapeutic interventions.

The present study is novel because it examined dosage effects of MBSR across time of the intervention. This approach demonstrated that dispositional mindfulness significantly increases as a function of meditation frequency and dosage. Importantly, increases in dispositional mindfulness were associated with reductions in trauma, grief, and sleep difficulty. Increases in dispositional mindfulness may also potentially help individuals transform their *relationship* with their grief. That is, it is postulated that because of potential feeling of guilt or a sense of betrayal, victims of gun violence and generalized trauma may not want to forget, or be distracted away from, or even feel better about the source of their grief. Rather, they are motivated to seek ways to carry on in their lives by altering the contextualization of their moment to moment experience. This is where the individual can *hold* mindful awareness of their loved one in their arising momentary experiences without allowing the corresponding trauma, grief, and dysphoria to obliterate said awareness. Thus, the individual is postulated to allow oneself to grieve and mourn without experiencing the health-debilitating effects of constant stress and depression that is prevalent in individuals experiencing this level of trauma and loss.

### Limitations and Future Research

The results of this nonrandomized, uncontrolled pilot trial on victims of gun violence should be interpreted with caution. The large effect sizes observed in the present study may simply be a reflection of nonspecific interventional factors corresponding to social support effects of participating in a behavioral intervention with a cohort of individuals that are sharing similar traumatic experiences (Foy et al., 2001). We also cannot rule out that elapsed time spent across the intervention, the therapeutic innervation of the mindfulness teachers, the multimodal aspects of participating in a mindfulness intervention, and/or other nonspecific or demand characteristics explain our results. Furthermore, all self-reported data were collected multiple times which may have increased common methods bias (Podsakoff et al., 2012). However, the temporal delay between each assessment time point (e.g., 5 weeks) reduces the prevalence of common methods bias (Jakobsen & Jensen, 2015). Importantly, according to the Centers for Disease Control and Prevention (2021), young Black men are 20 times more likely to be killed by a gun than White males of the same age group. The participants in the present study were 75% White. This is a significant limitation to the generalizability of the present findings. Thus, future clinical trials examining this question should include a more representative sample.

Further, we predict that future studies that engage multimodal aspects of present-centered savoring may be more effective at reducing trauma in this population (Garland et al., 2016). Recent accounts have demonstrated that mindfulness increases positive reappraisal processes to promote well-being (Garland, 2021; Garland et al., 2017; Harp et al., 2022). Others have described a so-called uncoupling (i.e., *Anatta*) between self-appraisal and arising sensory experience to facilitate a non-reactive meta-cognitive stance that alleviates suffering (Buddhadasa, 1990; Grant et al., 2011). Thus, based on converging and emerging mechanistic research, we predict that mindfulness improves trauma by attenuating self-referential processing and reflexive emotional reactivity to cultivate a more hedonically aligned subjective experience (Zeidan et al., 2014). Future assessments of mindfulness on victims of gun violence should be randomized, placebo-controlled, and engage stakeholders that reflect the prevalence of victims of gun violence. Nevertheless, this project is a first step in demonstrating the feasibility and efficacy of alleviating negative symptomology in victims of gun violence.

## Data Availability

We will provide deidentified data for this project in the very near future.
